# Spatiotemporal Analysis of Osteoblast Morphology and Wnt Signal‐Induced Osteoblast Reactivation during Bone Modeling in Vitro

**DOI:** 10.1002/jbm4.10689

**Published:** 2022-10-21

**Authors:** Naoki Tsuji, Tomoaki Sakamoto, Kazuto Hoshi, Atsuhiko Hikita

**Affiliations:** ^1^ Department of Sensory and Motor System Medicine, Graduate School of Medicine The University of Tokyo Tokyo Japan; ^2^ Department of Tissue Engineering The University of Tokyo Hospital Tokyo Japan

**Keywords:** ANABOLICS, BONE MODELING AND REMODELING, BONE MATRIX, OSTEOBLASTS, Wnt/β‐catenin/LRPs

## Abstract

Bone nodule formation by differentiating osteoblasts is considered an in vitro model that mimics bone modeling. However, the details of osteoblast behavior and matrix production during bone nodule formation are poorly understood. Here, we present a spatiotemporal analysis system for evaluating osteoblast morphology and matrix production during bone modeling in vitro via two‐photon microscopy. Using this system, a change in osteoblast morphology from cuboidal to flat was observed during the formation of mineralized nodules, and this change was quantified. Areas with high bone formation were densely populated with cuboidal osteoblasts, which were characterized by blebs, protruding structures on their cell membranes. Cuboidal osteoblasts with blebs were highly mobile, and osteoblast blebs exhibited a polar distribution. Furthermore, mimicking romosozumab treatment, when differentiated flattened osteoblasts were stimulated with BIO, a GSK3β inhibitor, they were reactivated to acquire a cuboidal morphology with blebs on their membranes and produced more matrix than nonstimulated cells. Our analysis system is a powerful tool for evaluating the cell morphology and function of osteoblasts during bone modeling. © 2022 The Authors. *JBMR Plus* published by Wiley Periodicals LLC on behalf of American Society for Bone and Mineral Research.

## Introduction

Bone homeostasis is regulated by the continuous interplay between bone formation by osteoblasts and bone resorption by osteoclasts.^(^ ^1^ ^)^ Osteoblasts secrete extracellular matrix proteins, such as type 1 collagen, osteopontin, and osteocalcin.^(^ ^2^, ^3^ ^)^ Osteoblast lineage cells are divided into osteoprogenitor cells, preosteoblasts, and osteoblasts during bone development. Subsequently, some osteoblasts become osteocytes, which are embedded within the bone matrix they produce, or bone‐lining cells (BLCs), which are found on the bone matrix.^(^ ^2^, ^4^ ^)^ BLCs are quiescent cells with a flattened morphology that cover the cortical endosteal surface.^(^ ^5^ ^)^ BLCs were reported to be flattened by sclerostin, which inhibits Wnt signaling and is secreted by osteocytes.^(^ ^6^ ^)^ It has been suggested that teriparatide, a parathyroid hormone used to treat patients with severe osteoporosis, and romosozumab, an antisclerostin antibody, reactivate BLCs and contribute to bone formation.^(^ ^7^, ^8^, ^9^ ^)^ However, there are no reports directly visualizing the reactivation of BLCs.

In vitro models of bone nodule formation by differentiating osteoblasts are used to evaluate the osteogenic capacity of cells.^(^ ^3^, ^10^, ^11^ ^)^ It has been shown that during bone nodule formation, the morphology of osteoblasts changes to an osteocyte‐like morphology when they are embedded within the bone matrix and that the ultrastructure of the formed bone matrix exhibits a woven bone architecture.^(^ ^3^ ^)^ The resemblance of the structure of bone nodules to native bones suggests that analyses of the bone nodule formation process may provide a deeper understanding of bone modeling in vivo, although the relevance of the in vitro bone nodule formation to in vivo bone modeling has not been proved. Nevertheless, the details of osteoblast morphology and behavior during bone nodule formation are poorly understood because it is difficult to quantify matrix production and observe the interior of the matrix under living conditions. To address this issue, we previously reported the application of two‐photon microscopy under living conditions, and we found that cells embedded inside the bone nodule had osteocyte‐like dendritic structures and that the cuboidal morphology of osteoblasts on the bone matrix changed to a flattened morphology.^(^ ^12^ ^)^ However, the details of the spatiotemporal cell behavior and morphological changes of osteoblasts remain unclear.

In this report, we quantitatively analyze the changes in osteoblast morphology over time during bone nodule formation by image analysis, and we analyze the changes in osteoblast morphology and matrix production when Wnt signaling is activated in differentiated and flattened osteoblasts.

## Materials and Methods

### Mice

All the animal experiments were approved by the animal experiment committee of the University of Tokyo Graduate School of Medicine (P15‐019 and P19‐114). C57BL/6‐Tg (CAG‐EGFP) mice were purchased from SLC (Japan SLC, Shizuoka, Japan).

### Preparation of primary osteoblasts

Calvarial osteoblasts were isolated as previously described.^(^ ^12^, ^13^ ^)^ Briefly, Calvariae were isolated from newborn mice at 0 to 7 days of age, and the soft tissues that adhered to their surface were scraped away with cell scrapers. Parietal bones were resected and incubated at 37°C for 20 minutes with shaking in digestion medium (3.5 ml α‐MEM [Life Technologies, Tokyo, Japan], 100 μg/ml collagenase P [Roche Diagnostics Japan, Tokyo, Japan], and 88 μl of 0.05% trypsin/EDTA [Life Technologies] for one calvaria). Then the bones were finely diced in double‐strength digestion medium (800 μl *α*‐MEM, 200 μg/ml collagenase *P*, and 20 μl of 0.05% trypsin/EDTA for one calvaria) and incubated at 37°C for 15 minutes with shaking. After incubation, 4 ml *α*‐MEM (supplemented with 100 U/ml penicillin, 100 μg/ml streptomycin, and 15% FBS) was added to suppress the enzymic activity.

### Cell culture

Calvarial osteoblasts were cultured as previously described with some modifications.^(^ ^12^, ^13^ ^)^ Briefly, 5.5 × 10^5^ primary osteoblasts were seeded in 60‐mm dishes to observe living cells using two‐photon microscopy. For 2–4 days, cells were cultured in α‐MEM supplemented with 100 U/ml penicillin, 100 μg/ml streptomycin, and 15% FBS. The day when the cells reached confluence was defined as Day 0. The cells were cultured in the following conditions: α‐MEM supplemented with 100 U/ml penicillin, 100 μg/ml streptomycin, 10% FBS, 100 μg/ml ascorbic acid (012–04802: Wako Pure Chemical Industries, Osaka, Japan), 5 mM β‐glycerophosphate disodium salt hydrate (G9422: Sigma‐Aldrich, St. Louis, MO, USA), and 0.1 μM cFMS Receptor Inhibitor II (Santa Cruz Biotechnology, USA). cFMS Receptor Inhibitor II was added to suppress the effects of macrophage lineage cells that might be contained in the primary osteoblasts collected. Images were obtained from multiple regions of interest (ROIs) in a single dish. To avoid contamination, the cells were washed twice with PBS before fresh medium was added. BIO (Selleck Biotech, USA) was diluted in ethanol and used at 1 μM.

### Staining of cultured cells

Cells were seeded in four‐well chamber slides (Chamber Slide II, IWAKI, Japan) at 5.0 × 10^4^ cells per well, incubated for the indicated numbers of days, and then fixed in 4% paraformaldehyde at 4°C for 15 minutes. Phalloidin‐Fluorescein (Wako, Osaka, Japan) was diluted in 0.05% Tween with PBS(−) and used to stain the cells for 20 minutes at room temperature. After washing with PBS, the cells were stained with 4,6‐diamidino‐2‐phenylindole (DAPI) (Dojindo, Kumamoto, Japan) for 15 minutes at room temperature.

### Image acquisition

The site of bone matrix production was confirmed on Day 7 with fluorescence and phase‐contrast microscopy (Leica DMi8, Leica, Germany), and the bottom of the dish was marked with a red pen (Hi‐Mckee, ZEBRA, Japan). Two‐photon excited fluorescence images and second harmonic generation (SHG) images were acquired using a multiphoton confocal microscopy system (A1R + MP, Nikon) with an excitation laser (Mai Tai eHP, wavelengths: 680–950 nm; repetition rate: 80 MHz; pulse width: 70 fs, Spectra‐Physics, Tokyo, Japan) and a water‐immersion objective lens (CFI75 Apo 25 × W MP, numerical aperture: 1.1, Nikon). The emission filters used were as follows: 492 nm shortpass for SHG and 525/50 nm bandpass for EGFP. To observe the same ROI, a red pen was used to mark the side of the dish, and the dish was placed so that the mark on the bottom of the stage incubator matched the mark that had previously been placed on the bottom of the stage incubator (TOKAI HIT, Japan) (Fig. S1a–c). Observations of osteoblast and matrix changes were made with galvanometer mirrors at *Z*‐steps of 1 μm. In the observation of osteocyte‐like dendrites, the images were captured at Zoom 3× with 0.45 μm *Z*‐steps. To observe bleb and intracellular vesicles, images were captured with resonant mirrors at Zoom 4–8× and averaged 8×, and *Z*‐steps were taken at 1 μm. The four‐dimensional (4D) images of blebs were taken with a *Z*‐stack of 30 μm. For 6 to 10 hours of time‐lapse imaging, images were captured at an interval of 15 minutes, and for 58 to 74 hours of time‐lapse imaging, images were captured at an interval of 30–60 minutes. All images were captured at a wavelength of 950 nm.

### Image analysis

All the images were preprocessed by NIS Elements version 4.30.00 (NIKON). For morphometric analysis of osteoblasts, the acquired data were processed using a median filter and local contrast image processing. In SHG, noise was removed with a median filter. Lacuna structures were extracted by median filtering of the acquired SHG images, followed by local contrast, and then by median filtering to remove noise after extraction using regional minima. The bleb region was extracted from the 4D image from frame‐by‐frame differences. The XYZ misalignment of the data acquired by time lapse was adjusted manually. Preprocessed images were analyzed using IMARIS version 8.3.1 (Oxford Instruments, Abingdon, UK). Bleb extraction in three dimensions was performed with subtractive minimum intensity in time. The bleb area and duration were quantified by NIS Elements version 4.30.00 (NIKON). The IN/ON and OUT osteoblasts were manually selected by two observers or projected into MATLAB R2022a (MathWorks, USA) of the SHG quantitative data using Imaris Reader^(^ ^14^ ^)^ and then, after the vertices of the boundaries were calculated using the boundary function, the numbers of osteoblasts present within the boundaries were calculated using the inpolygon function, and those present above the lowest point of the SHG height were defined as IN/ON. Delaunay triangulation was performed using the Delaunay triangulation function in MATLAB R2022a (MathWorks, USA) from osteoblast data quantified by IMARIS version 8.3.1 (Oxford Instruments) to calculate the area of the triangles.

### Real‐time PCR (RT–qPCR)

Total RNA was isolated from the collected samples using ISOGEN (Nippon Gene Co., Tokyo, Japan) according to the instructions provided by the manufacturer. RNA was reverse transcribed using the PrimeScript™ reagent Kit (TAKARA Bio Inc., Tokyo, Japan). Gene expression was measured using the THUNDERBIRD® Next SYBR® qPCR Mix (TOYOBO, Tokyo, Japan). Real‐time qPCR was performed on a 7500 Fast Real‐Time PCR System (Applied BioSystems, CA, USA) to measure the relative gene expression levels, which were calculated using the ΔΔCT method. Table S1 lists the primers used for real‐time qPCR (Table S1). Primer specificity was confirmed using GGRNA version 1.^(^ ^15^ ^)^

### Statistical analysis

MATLAB R2022a (MathWorks, USA) was used to graph the data and to perform statistical analyses. In Fig. 6, the error bars indicate the mean ± SE. All other data are expressed as the mean ± SD. After one‐way ANOVA, Tukey's test was performed as a post hoc analysis. After the normality of the data was tested with the Kolmogorov–Smirnov test, the correlation coefficient was calculated with Spearman's rank correlation coefficient. Rho values of 0 ≤ and <0.2, 0.2 ≤ and <0.4, 0.4 ≤ and <0.6, and 0.6 ≤ and <0.8 were considered to indicate little, weak, moderate, and strong correlations, respectively. A paired *t*‐test was performed for the corresponding two‐group comparison. A two‐sample *t*‐test was performed for two group comparisons with no correspondence. Statistical significance (*p* value) was set at 5%, where *p* < 0.05 was the level at which statistical significance was defined.

## Results

### Live cell imaging of bone nodule formation in the same regions over time

**Fig. 1:**
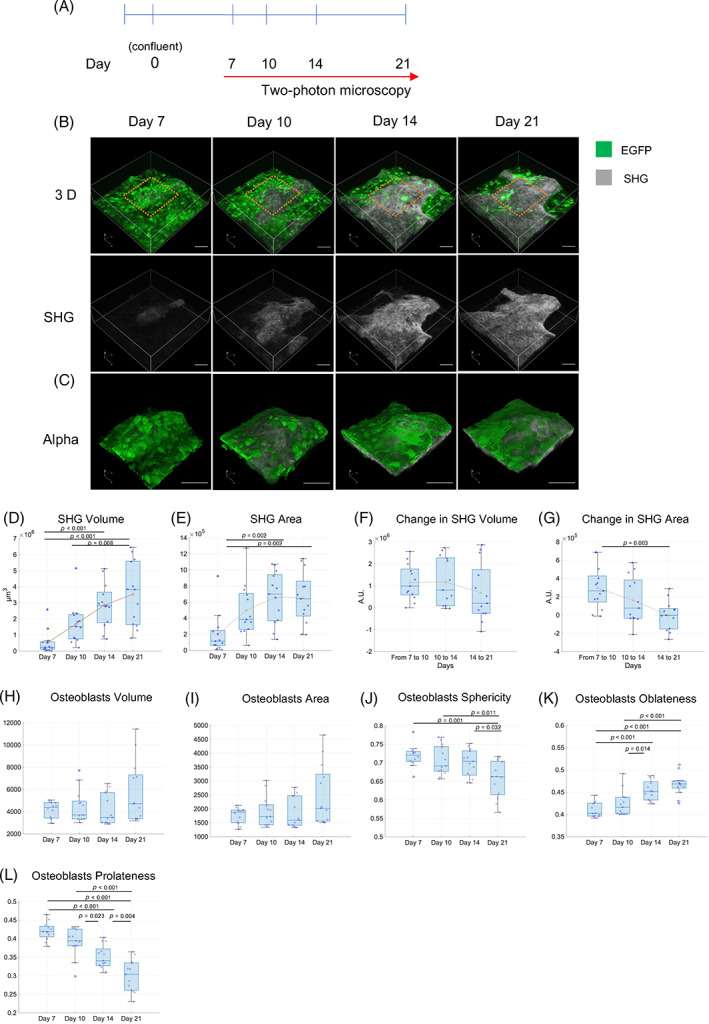
Live cell imaging of bone nodule formation over time. (*A*) Schematic of experimental design. (*B*) Live cell imaging of bone nodule formation over time using two‐photon microscopy. (*C*) Alpha rendering images in cropped areas indicated in (*B*) (d)–(g). Quantification of parameters for SHG. (*D*) Change in SHG volume over time. (*E*) Change in SHG area over time. (*F*) Change in volume since last observation. (*G*) Change in area since last observation. Orange points indicate average values. AU, arbitrary unit. (*H*–*I*) Quantification of parameters for osteoblasts. (*H*) Osteoblast volume. (*I*) Osteoblast area. (*J*) Osteoblast sphericity. (*K*) Osteoblast oblateness. (*L*) Osteoblast prolateness. In (*D*–*I*) were plotted the mean values of each ROI. (*D*–*L*) *N* = 13 from three independent experiments. One‐way ANOVA with Tukey's post hoc multiple comparison test. Scale bar: 100 μm.

Primary osteoblasts derived from neonatal CAG‐EGFP mice were cultured under differentiation conditions. Nodule formation by osteoblasts was confirmed by phase contrast microscopy on Day 7 (Fig. S2a). The bottom surface of the dish was marked with a red pen (Fig. S2b), and changes over time in the same area were observed with a two‐photon microscope using the markings as a landmark (Fig. 1*A*, Fig. S2c). Two‐photon microscopy enables the observation of deep areas of biological tissues and the detection of collagen fibers by SHG without staining.^(^ ^16^, ^17^ ^)^ As shown in Fig. 1*B*, we were able to observe an increase in the collagen matrix over time. In addition, the increase in the collagen volume and area were quantitatively evaluated using IMARIS software (Fig. 1*D*, *E*). On the other hand, the increase in the collagen matrix was shown to gradually decrease in the late stages (Fig. 1*F*, *G*).

In addition to the change in matrix volume, the shape of the osteoblasts on the bone nodules changed over time from a cuboidal to a flattened morphology (Fig. 1*C*). Quantitative analysis of osteoblast morphology showed that osteoblasts outside the bone matrix (OUT) did not significantly change their morphology (Fig. S3), whereas the morphology of the osteoblasts in and on the bone matrix (IN/ON) became less spherical and prolate and more oblate over time (Fig. 1*J*–*L*). There were significant differences between IN/ON osteoblasts and OUT osteoblasts in volume and area on all the observation days (Fig. S4a, b). Sphericity was significantly different on Day 21 (Fig. S4c). The results of oblateness and prolateness, which are measures of flatness, revealed significant differences in oblateness except for Day 10 (Fig. S4d) and significant differences in prolateness except for Day 14 (Fig. eS4). These results show that osteoblasts on the bone matrix change their morphology from cuboidal to flat.

### Spatiotemporal analysis of osteoblast morphology and matrix production during bone nodule formation

**Fig. 2:**
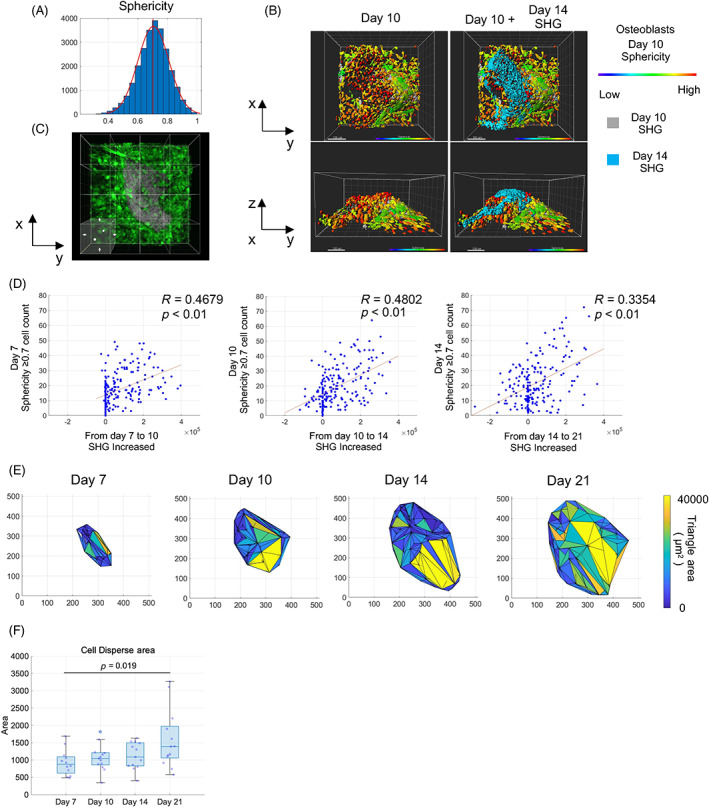
Crowding, cuboidal osteoblasts contribute to osteogenesis. (*A*) Histogram of osteoblast sphericity. (*B*) Osteoblasts shown with heatmap color and SHG of Days 10 and 14. (*C*) Division of sub‐ROIs in observation field of view. (*D*) Scatter plots of number of osteoblasts with high sphericity and increase in SHG. N = 208 from three independent experiments. R indicated Spearman's rank correlation coefficient. (*E*) Representative images of osteoblast density calculated by Delaunay triangulation. (*F*) Boxplot of mean areas of triangles. *N* = 13 from three independent experiments. One‐way ANOVA with Tukey's post hoc multiple comparison test. Scale bar: 25 μm.

It is widely accepted that cuboidal osteoblasts are the most active osteogenic cells. We focused on the sphericity of osteoblasts and its spatiotemporal relations to matrix production. We evaluated the histogram of osteoblast sphericity and found that the average was 0.7026 (Fig. 2*A*). We defined osteoblasts with a sphericity of 0.7 or above as cells with high sphericity. When Day 14 SHG data were merged with Day 10 osteoblasts and SHG of the same ROI, the increased area of the matrix overlapped with the areas of high sphericity in Day 10 osteoblasts (Fig. 2*B*). To confirm the topological correlation between the sphericity of osteoblasts and matrix deposition, the field of observation was divided into 16 sections for random evaluation (subROIs) (Fig. 2*C*), and the number of cells with high sphericity in the subROIs and the changes in the volume of SHG were plotted.

The number of cells with high sphericity on Days 7 and 10 moderately correlated with the degree of change in SHG from Day 7 to Day 10 and from Day 10 to Day 14, respectively (Fig. 2*D*). The number of osteoblasts on Day 14 and matrix increase from Day 14 to Day 21 showed a weak positive correlation (Fig. 2*D*), and the reason for this weak correlation seems to be the presence of osteocytes among cells with high sphericity and a decrease in osteogenic potential over time. The number of cells with high sphericity in the subROIs increased up to Day 14 and then decreased on Day 21 (Fig. S5a–c). Next, to quantify the degree of cell crowding over time, Delaunay triangulation was performed on the IN/ON osteoblasts, and the mean area of the triangles was calculated (Fig. 2*E*). The mean area of the triangle showed an increase over time (Fig. 2*F*).

To rule out the influence of osteocytes, lacuna structures were extracted from the SHG images by image processing (Fig. S6a). The extracted lacuna structures were constructed in three dimensions, and spot detection was performed (Fig. S6b). The spot‐detected data were used to perform Delaunay triangulation and calculate the mean area of the triangles. The analysis was performed beginning on Day 10 because few lacuna structures were observed on Day 7. The mean triangular areas of lacunae did not change significantly over time (Fig. S6c, d). These results suggest that the area of the Delaunay triangles increased due to the altered density of osteoblasts on the bone matrix.

### Cuboidal and crowded osteoblasts had many blebs

**Fig. 3:**
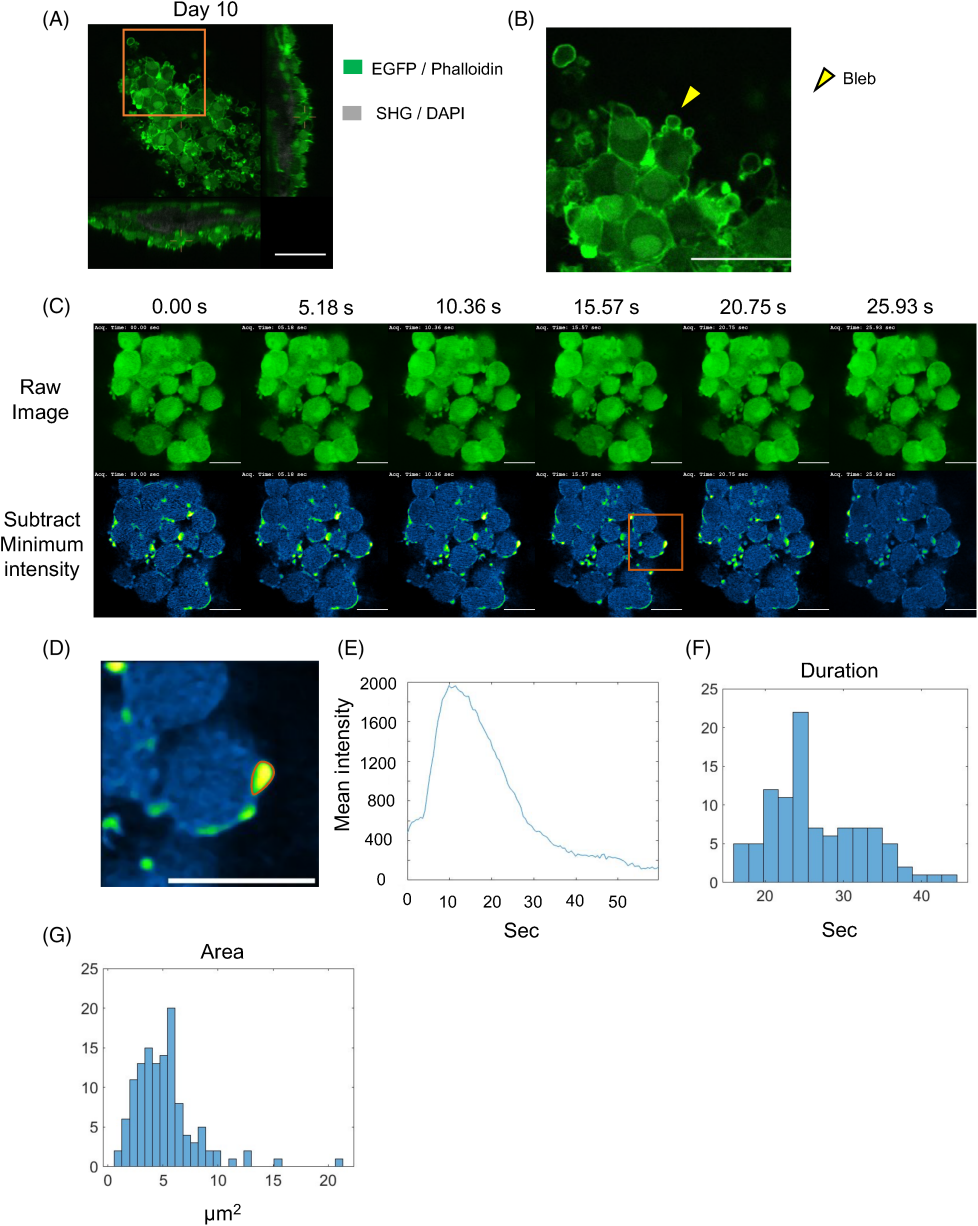
Cuboidal osteoblasts had many blebs. (*A*) Representative images of Day 10 osteoblasts after staining with phalloidin and 4,6‐diamidino‐2‐phenylindole. (*B*) Bleb formation observed in osteoblast membranes (yellow arrowhead indicates bleb). (*C*) Time‐lapse images of cuboidal osteoblasts and images obtained by subtracting minimum intensity from acquired images and changing color of luts. (*D*) Representative image cropped from (*C*). (*E*) Mean intensity of bleb regions. (*F*) Histogram of blebbing duration (*N* = 82). (*G*) Histogram of maximum area of observed blebbing areas (*N* = 123). Scale bar: 25 μm.

**Fig. 4:**
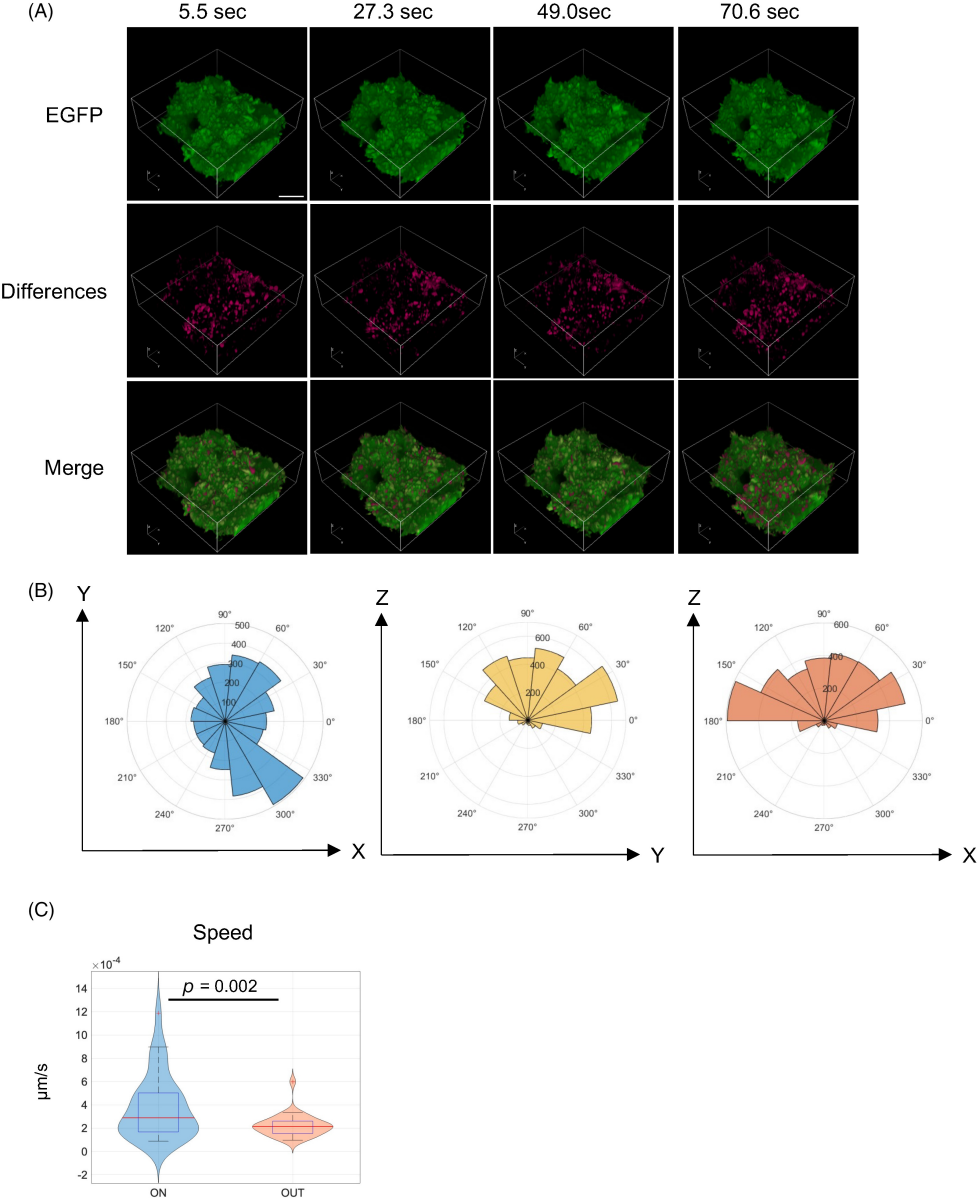
Blebs have polarity, and cuboidal osteoblasts have high motility. (*A*) Representative images of time lapse in four dimensions and images extracted from frame‐by‐frame differences and their merged images. (*B*) Polar plots of distribution of bleb in *xy*, *zy*, and *zx* planes. (*C*) Violin plots of speed of cuboidal osteoblasts on bone matrix and adherent osteoblasts on dish, paired *t*‐test. Scale bar: 25 μm.

Further observations of osteoblast morphology were performed using two‐photon microscopy after staining with phalloidin and DAPI. On Day 10, osteoblasts on the bone matrix (surface) had a cuboidal morphology, whereas the morphology of osteoblasts that could penetrate the matrix (superficial) and osteoblasts within the bone matrix (deep) changed to one with osteocyte‐like dendritic processes (Fig. S7a). On Day 14, the morphology of surface osteoblasts had changed to on with an elongated cytoskeleton, and deep osteoblasts communicated with each other through dendrites (Fig. S7b, c). Furthermore, osteocyte dendrites were found to be in contact with each other and with osteocytes and osteoblasts on the matrix (Fig. S7d, e). We found that cuboidal and crowded osteoblasts, in which osteogenesis actively occurred, had protruding structures on their cell membranes (Fig. 3*A*, *B*). These protruding structures, known as blebs, are required for the amoeboid migration that cancer cells^(^ ^18^ ^)^ and immune cells^(^ ^19^, ^20^ ^)^ perform as they invade tissues. Bleb formation in osteoblasts has been reported to involve P2X7 receptors.^(^ ^21^ ^)^ However, it remains unclear whether blebs are involved in bone formation. Time‐lapse imaging showed that cuboidal osteoblasts were actively blebbing (Fig. 3 *C*, Video S1). When the minimum intensity was subtracted from the acquired image data, blebs could be clearly distinguished (Fig. 3*C*, *D*). The average duration of the blebs was approximately 27 seconds, and their average area was approximately 5 μm^2^ (Fig. 3*E*–*G*). We next examined the directivity of the blebs in three dimensions. We analyzed each frame for no more than 27 seconds. Blebs could be clearly distinguished by extracting differences for each frame from the acquired image data (Fig. 4 *A*, Video S2). To examine the polarity of a bleb, the angle from the center of the osteoblast was calculated based on the coordinates of the spot‐detected blebs. We found that blebs were distributed throughout the osteoblasts in the XY plane, but very few were found in the XZ and YZ planes below the osteoblasts (Fig. 4*B*). This indicated that blebs were less likely to occur on surfaces involved in cell–cell contact or cell–matrix contact. In addition, at 9 and 74 hours of time‐lapse imaging, cuboidal osteoblasts showed high motility, with little apoptosis (Video S3, S4). The rate of movement was significantly higher in osteoblasts on the bone matrix than in those outside the bone matrix (Fig. 4*C*). This indicated that cuboidal osteoblasts form many blebs on the cell membrane and have high motility.

### Differentiated flattened osteoblasts were reactivated by stimulation of Wnt signaling

**Fig. 5:**
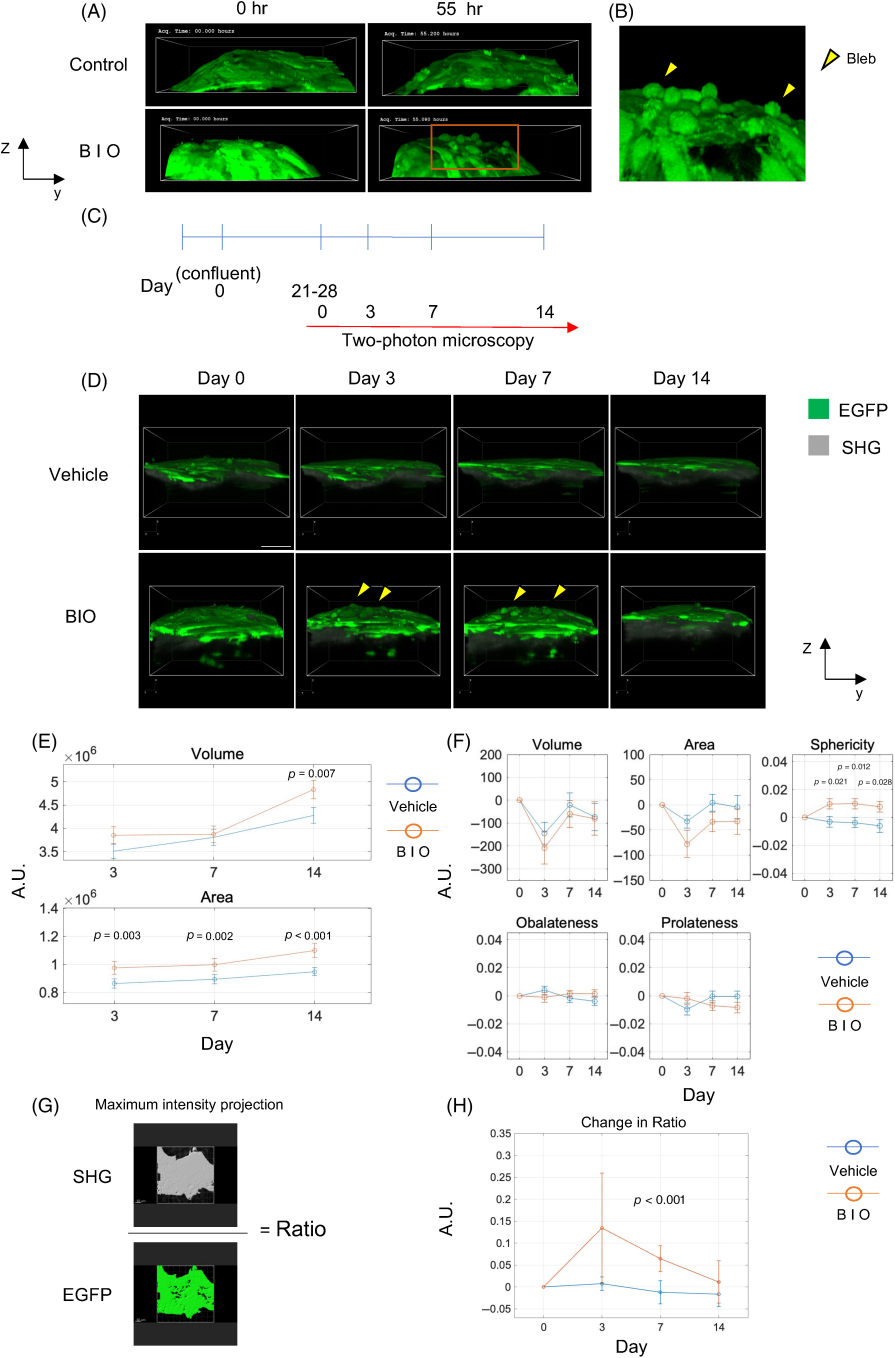
Time‐lapse observation of changes in bone nodules and osteoblast morphology induced by BIO. (*A*) Representative images of osteoblast morphological changes from 0 to 55 hours after BIO administration. (*B*) Cropped image from (*A*) of osteoblasts that acquired a cuboidal shape after BIO administration (yellow arrows indicate blebs). (*C*) Schematic of experiment to analyze effects of BIO administration in long term. (*D*) Representative images of osteoblast morphological changes in BIO and vehicle groups (yellow arrows indicate osteoblasts that acquired a cuboidal morphology). (*E*) Line plots with error bars of SHG increase by BIO administration. (*F*) Changes in IN/ON osteoblast morphology over time following BIO administration. (*G*) Binarization of SHG and osteoblasts by IMARIS for maximum intensity projection. (*F*) SHG/EGFP ratio changes over time. *N* = 20 from five independent experiments. AU, arbitrary unit. Two‐sample *t*‐test (*E*, *F*, *H*). Data shown as means ± SE. Scale bar: 50 μm.

We observed that the morphology of osteoblasts on bone nodules changes from cuboidal to flat following increases in matrix production. The change in osteoblasts to BLCs was thought to result from the inhibition of Wnt signaling by sclerostin from osteocytes.^(^ ^6^ ^)^ Romosozumab, an antisclerostin antibody used for the treatment of osteoporosis, is thought to activate BLCs and contribute to bone formation. Therefore, we examined whether differentiated and flattened osteoblasts could be reactivated when Wnt signaling was stimulated. We performed long‐term time‐lapse imaging to confirm whether osteoblast morphology was altered after treatment with BIO, a GSK3β inhibitor, in long‐term culture. In the BIO‐treated group, flattened osteoblasts were observed to change to cuboidal osteoblasts (Fig. 5 *A*, Video S5, S6). Furthermore, osteoblasts that changed to cuboidal morphology were observed to form blebs on their cell membranes (Fig. 5*B*). Next, we observed the changes in osteoblast morphology and matrix formation over time after BIO administration (Fig. 5*C*). In the BIO‐treated group, cuboidal osteoblasts were observed on Days 3 and 7. On Day 14, osteoblasts returned to a flattened morphology (Fig. 5*D*). Quantification of SHG showed a significantly increased volume on Day 14 compared to the vehicle group and a significantly increased area on all the indicated days after treatment (Fig. 5*E*). Quantification of the changes in IN/ON osteoblast morphology showed an increase in sphericity with BIO treatment (Fig. 5*F*). We examined whether BIO administration altered the percentage of osteoblast surface on the SHG due to a cuboidal change in osteoblast morphology. After converting the acquired image data to maximum intensity projection, SHG and osteoblasts were binarized by surface, and the ratios were calculated (Fig. 5*G*). The degree of change was plotted, and the results showed that the ratio increased on Days 3 and 7 in the BIO‐treated group and returned to the same level as before treatment on Day 14 (Fig. 5*H*). RT–qPCR showed that ALP expression was increased on Days 3 and 14 and that Runx2 expression was increased on Day 14 after BIO administration (Fig. S8a–d).

## Discussion

We established a spatiotemporal analysis system for evaluating osteoblast morphology and matrix production during the formation of bone nodules by osteoblasts using two‐photon microscopy. Two‐photon microscopy enabled the detection of collagen without staining^(^ ^16^, ^17^ ^)^ and observation of changes in osteoblast morphology and matrix production over time. Mature osteoblasts are known to form clusters with cuboidal morphology on the surface of bones.^(^ ^22^ ^)^ Our findings showed that areas in which osteogenesis was actively occurring were densely populated with cuboidal‐shaped osteoblasts, similar to cluster patterns, even during bone nodule formation. Osteoblasts were observed to develop osteocyte‐like dendrites while embedding in the bone matrix, and the dendrites contacted cells on the surface of the bone matrix and formed connections between osteocytes, as was reported previously.^(^ ^4^, ^23^ ^)^ Osteoblasts that remain on the bone matrix are known to change their morphology to become quiescent BLCs.^(^ ^5^, ^24^, ^25^ ^)^ We observed that the morphology of the remaining osteoblasts on the bone matrix changed from cuboidal to a flattened, bone‐lining cell‐like morphology.

Amoeboid migration is a mode of migration by which cancer cells and immune cells migrate within the extracellular matrix and are known to generate protruding structures called blebs on the cell membrane.^(^ ^26^, ^27^ ^)^ To induce blebbing in vitro, cells were embedded in extracellular matrix and observed,^(^ ^27^, ^28^ ^)^ whereas spontaneous blebbing was observed in osteoblasts. In reports about osteoblasts, Panupinthu, Nattapon, et al. reported that lysophosphatidic acid was produced by P2X7 receptors via phospholipase in primary osteoblasts and caused blebbing.^(^ ^21^ ^)^ Baldini, Giovanna, et al. reported that the specific inhibition of the RNA expression of secreted protein acidic and rich in cysteine (SPARC) significantly reduced the blebbing ability of colchicine‐treated HOBIT cells, which are a human osteoblast‐like cell line, and that SPARC played a direct role in cell morphology dynamics during cytoskeletal reorganization.^(^ ^29^ ^)^ Mizoguchi et al. showed fluorescent pseudo‐confocal microscopy images of the endosteal surface of osteocalcin (Bglap)‐GFP mice at postnatal Day 21 (3 weeks of age). These images showed mature osteoblasts with the bleblike structures in vivo.^(^ ^30^ ^)^ However, it remains unclear whether osteoblast blebbing contributes to the osteogenic process. Osteoblasts are known to secrete matrix vesicles to calcify collagen fibers. Budding has been proposed as the mode of matrix vesicle secretion.^(^ ^31^, ^32^ ^)^ Considering the structural similarity of buds that eventually formed blebs that we observed in this study, blebbing could be involved in the secretion of matrix vesicles. However, some researchers have recently suggested that matrix vesicles are exosomes^(^ ^33^, ^34^ ^)^ that are not secreted by budding but by exocytosis.^(^ ^35^, ^36^ ^)^

We observed that blebs did not form on the cell membranes of osteoblasts that were attached to other osteoblasts or bone matrix, indicating that the blebs of osteoblasts had polarity. It has been reported that osteoblasts communicate with each other via gap junctions and transfer calcium ions.^(^ ^33^ ^)^ It was considered that the bleb polarity was caused by the lack of calcium ion influx from outside the cell, which is necessary for bleb formation,^(^ ^27^, ^37^ ^)^ at the gap junction site of osteoblasts. However, the cause of polarity in the blebs of osteoblasts requires further investigation.

BLCs are abundant on bone surfaces.^(^ ^5^, ^24^, ^25^ ^)^ Teriparatide, a parathyroid hormone used as an osteogenic agent in recent osteoporosis, and romosozumab, an antisclerostin antibody, have been reported to reactivate BLCs and contribute to bone formation.^(^ ^7^, ^8^, ^9^ ^)^ In our study, we observed that activation of the Wnt signal by BIO administration induced a change in the flattened morphology of BLC‐like cells to a cuboidal morphology on the bone matrix and an increase in the bone matrix 2 weeks after administration. In addition, on Day 14 after BIO administration, the cuboidal morphology of osteoblasts returned to the flat morphology they had exhibited before administration. This transient effect of BIO on osteoblast morphology is consistent with the fact that the osteogenic effect of romosozumab has been reported to decrease over time.^(^ ^8^ ^)^ Previous reports suggested that the decrease in osteogenic potential after long‐term treatment with romosozumab was due to a decrease in the reserve of quiescent BLCs that could be activated.^(^ ^7^, ^38^, ^39^ ^)^ In our study, the change in osteoblast morphology might have reflected a decrease in the number of BLC‐like cells remaining on the bone matrix, whereas the cells that were activated by BIO became embedded within the bone matrix or underwent apoptosis. Another possible explanation of the transient effect of BIO is a bone mechanostatic response that induces overexpression of counterregulatory molecules, such as Dickkopf‐related protein 1.^(^ ^8^, ^40^ ^)^ In addition, BIO is a pan‐GSK3α/β inhibitor and may act on kinases such as cdk5/p25, cdk2/cyclin a, and cdk1/cyclin b.

We previously reported an experimental system that recapitulated bone remodeling by coculturing osteoblasts and osteoclasts that had been induced to differentiate over a long period of time.^(^ ^12^ ^)^ We observed that when reversal cell‐like osteoblasts migrated into the bone resorption pits generated by osteoclasts, the flattened osteoblasts acquired a cuboidal shape. Our previous study and this study showed that osteoblasts on immature bone matrix in the early stages of bone formation and osteoblasts in the resorption pits generated by osteoclasts acquired a cuboidal morphology. This suggests that osteoblasts were flattened due to the suppression of Wnt signaling by the sclerostin secreted from osteocytes in the microenvironment and that osteoblasts mobilized in the resorption pits might have acquired a cuboidal shape due to the reduction in osteocyte numbers by osteoclast bone resorption.

In addition, in this study, blebbing of osteoblasts was observed only in osteoblasts on the bone matrix, a three‐dimensional scaffold, which may be due to their interaction with the extracellular matrix. During the process of bone nodule formation, the directed movement of osteoblasts toward the bone matrix may be influenced by the stiffness of the scaffold. Recently, mesenchymal stem cells (MSCs)) have been reported to sense the stiffness of the scaffold, which determines the direction of differentiation,^(^ ^41^ ^)^ and durotaxis has been reported to cause MSCs, neural crest cells,^(^ ^42^ ^)^ and fibroblasts^(^ ^43^ ^)^ to migrate according to the stiffness gradient of the scaffold. In osteoblasts, scaffold stiffness gradients have also been reported to be involved in osteogenic function.^(^ ^44^, ^45^ ^)^ Undifferentiated osteoblasts are known to have higher motility than differentiated osteoblasts.^(^ ^46^ ^)^ We hypothesize that it is the stiffness gradient of the scaffold that regulates changes in the behavior of osteoblasts during bone nodule formation.

**Fig. 6:**
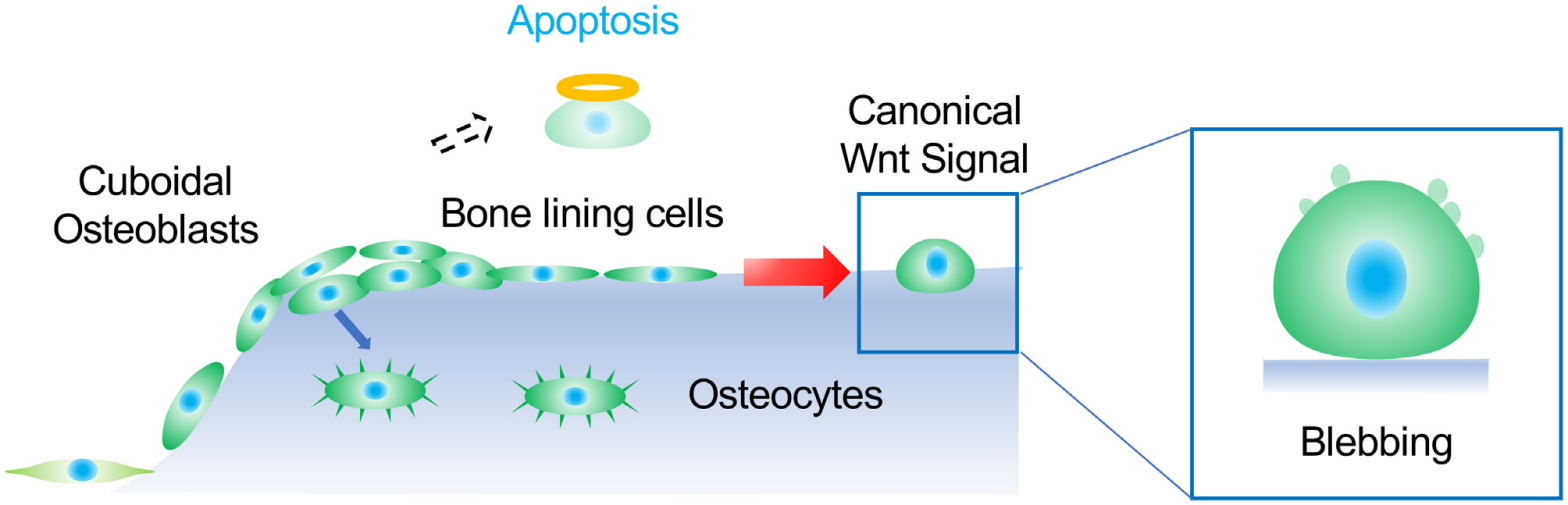
Schematic of osteoblast morphology and behavior during bone nodule formation. Osteoblasts had a crowded cuboidal morphology in the early stages of bone formation; some were embedded in the bone matrix and became osteocytes, and the remaining osteoblasts acquired a flattened BLC‐like morphology. When Wnt signaling was activated in the flattened osteoblasts, they again acquired a cuboidal shape. Cuboidal and reactivated osteoblasts had many blebs on their cell membranes.

In conclusion, we used two‐photon microscopy to observe the morphology and behavior of osteoblasts during bone nodule formation. Cuboidal osteoblasts had high motility and high osteogenic potential, and they formed blebs on their cell membrane. Osteoblasts with flat morphology, such as BLCs, acquired a cuboidal morphology when the Wnt signaling pathway was activated by BIO administration (Fig. 6). Reactivated osteoblasts possess many blebs, whose role in osteogenesis should be determined in future studies.

## Author Contributions

Naoki Tsuji and Atsuhiko Hikita contributed to the design of the study. Naoki Tsuji performed all the experiments described in this manuscript. Tomoaki Sakamoto provided technical support. Kazuto Hoshi and Atsuhiko Hikita supervised the studies and directed the interpretations of the results.

## Disclosures

Atsuhiko Hikita was affiliated with an endowed chair supported by FUJISOFT INCORPORATED (until October 31, 2020) and an endowed chair supported by CPC Corp., Kyowa Co., Kanto Chemical Co., and Nichirei Corp. (from July 1, 2021 to June 30, 2022) and is affiliated with the social cooperation program with Kohjin Bio Co. (from July 1, 2022).

### Peer Review

The peer review history for this article is available at https://publons.com/publon/10.1002/jbm4.10689.

## Supporting information


**Appendix S1** Supporting Information

**Video S1** Supporting Information

**Video S2** Supporting Information

**Video S3** Supporting Information

**Video S4** Supporting Information

**Video S5** Supporting Information

**Video S6** Supporting Information

## Data Availability

The data sets generated during the current study are available from the corresponding author (Atsuhiko Hikita) upon reasonable request.
