# Supplementary material for: Spatiotemporal Analysis of Osteoblast Morphology and Wnt Signal‐Induced Osteoblast Reactivation during Bone Modeling in Vitro
Source: JBMR Plus. 2022 Oct 21;6(11):e10689. doi: 10.1002/jbm4.10689 (PMC9664540; doi:10.1002/jbm4.10689)
Supplement: Supplementary file 1 — Appendix S1 Supporting Information [file JBM4-6-e10689-s005.pdf]

Supplementary Figure. 1.

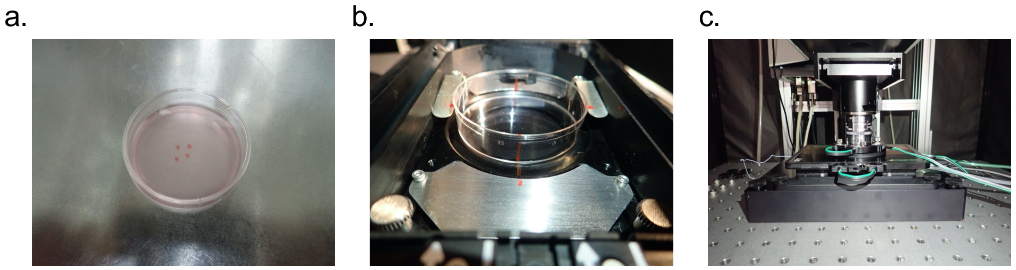

Methods to obtain the identical ROIs.
a.Marking of the observation site on the bottom of the dish.b. Matching dish side markings and stage incubator markings for observation of the same site.c. Setting at the observation.

Supplementary Figure. 2.

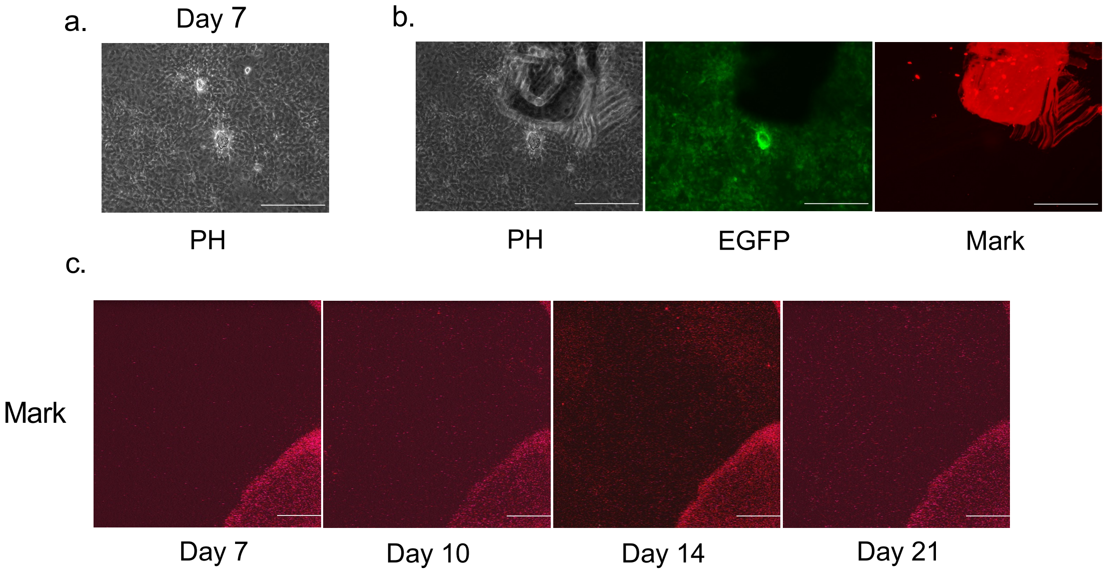

Marking of observation sites and tracking of the same sites over time
a. Day 7 phase contrast images of bone nodules. b. Marking on the bottom of the 60 mm dish.
c.Images of red markers on the bottom of the 60 mm dish on indicated time points.

Supplementary Figure. 3.

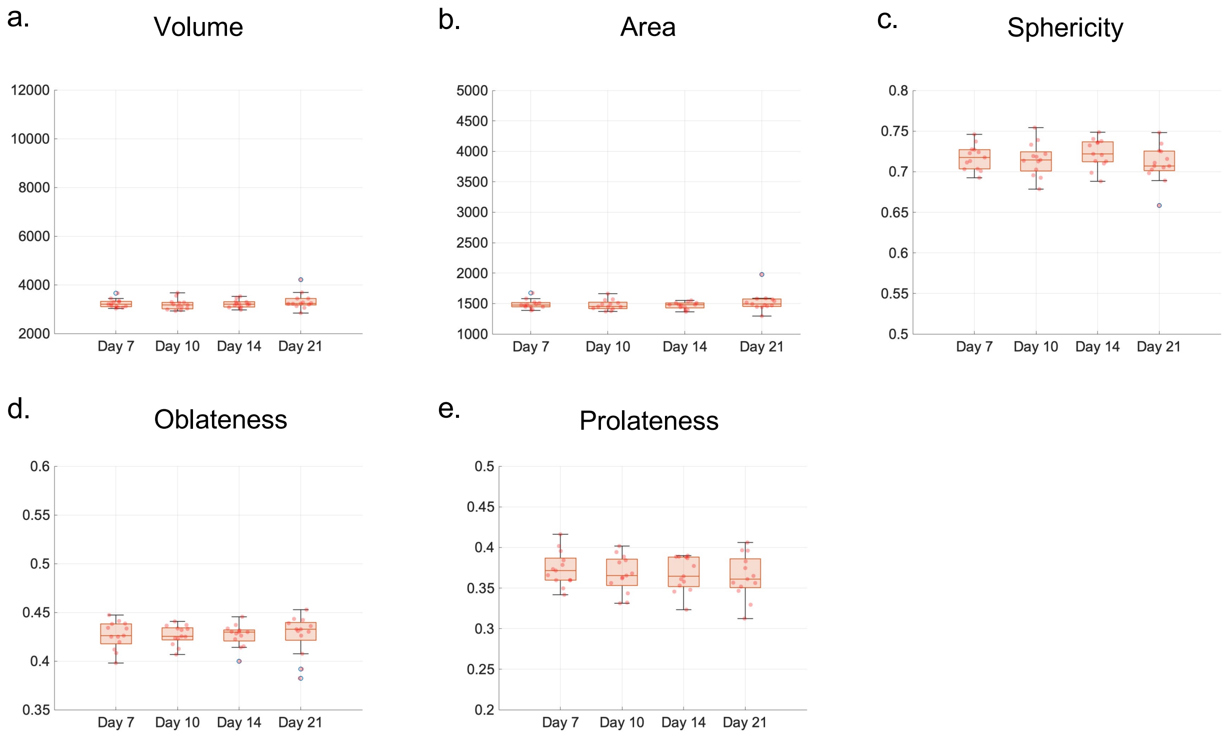

Morphometric quantification of osteoblasts outside the bone nodule
a.Volume. b.Area. c.Sphericity. d.Oblateness. e.Prolateness. A-e were plotted the mean value of each ROI.
N = 13 from three independent experiments.

Supplementary Figure. 4.

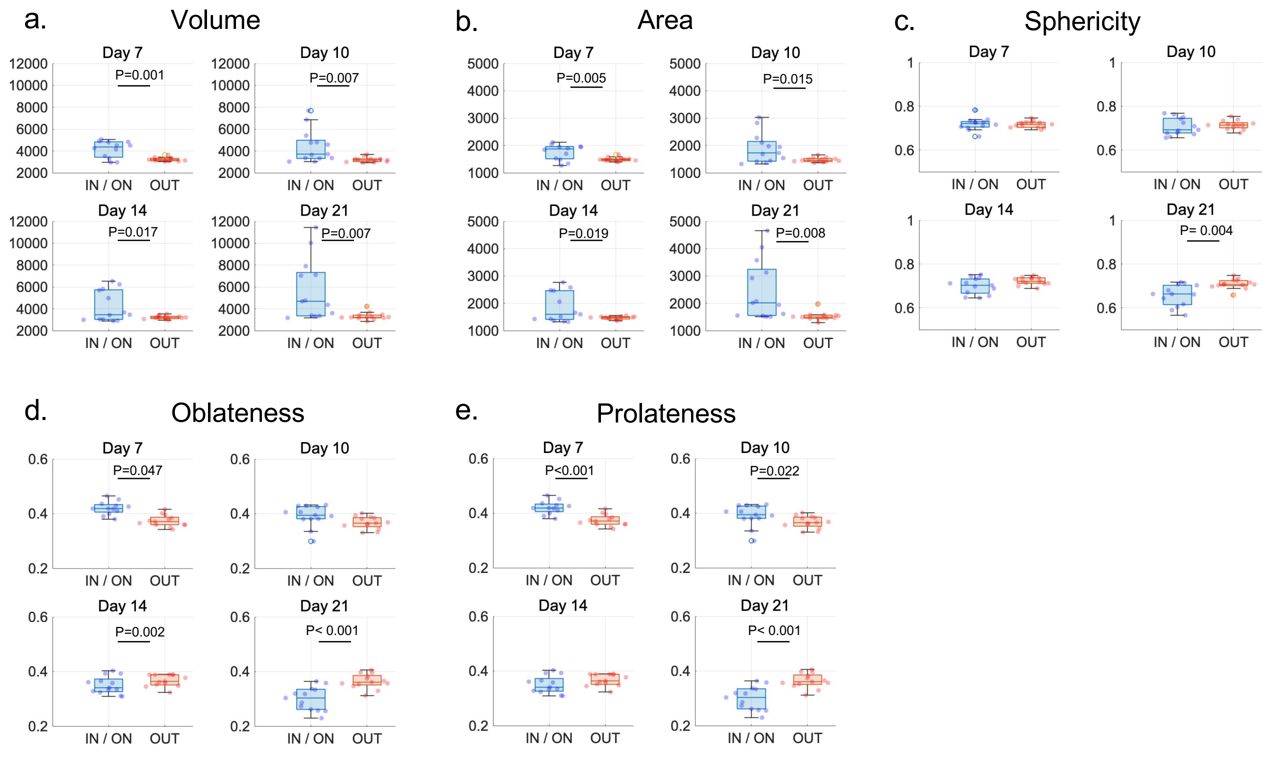

Comparison of osteoblast morphology IN/ON and OUT
a.Volume. b.Area. c.Sphericity. d.Oblateness. e.Prolateness. N = 13 from three independent experiments.,
One-way ANOVA with Tukey’s post-hoc multiple comparison test.

Supplementary Figure. 5.

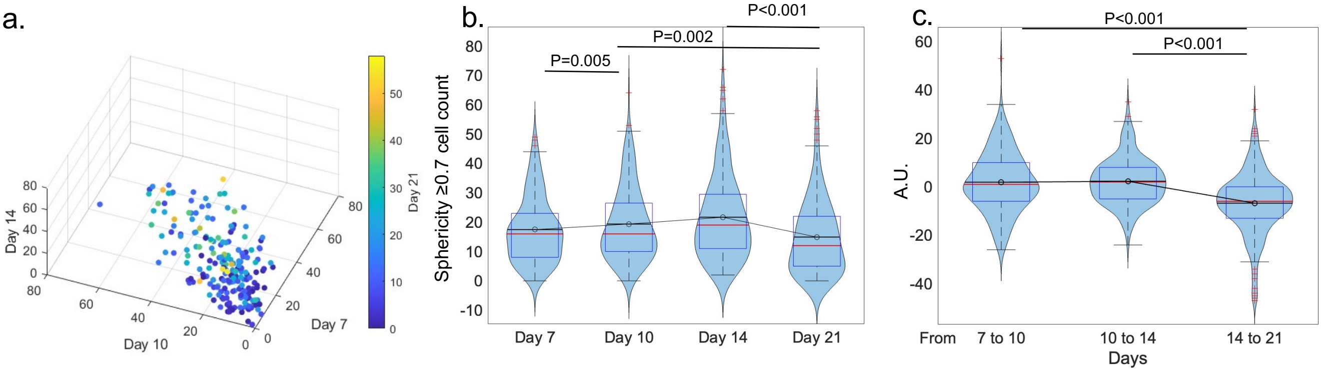

Changes over time in the number of high sphericity osteoblasts in subROIs
a.Visualization of data distribution with 4D plots. b. Violin plots of cell number changes in subROIs.
c. Change in cell count since last observation. Black points indicated the average values. A.U.: arbitrary unit. ,
N = 208 from three independent experiments. One-way ANOVA with Tukey's post-hoc multiple comparison test.

Supplementary Figure. 6.

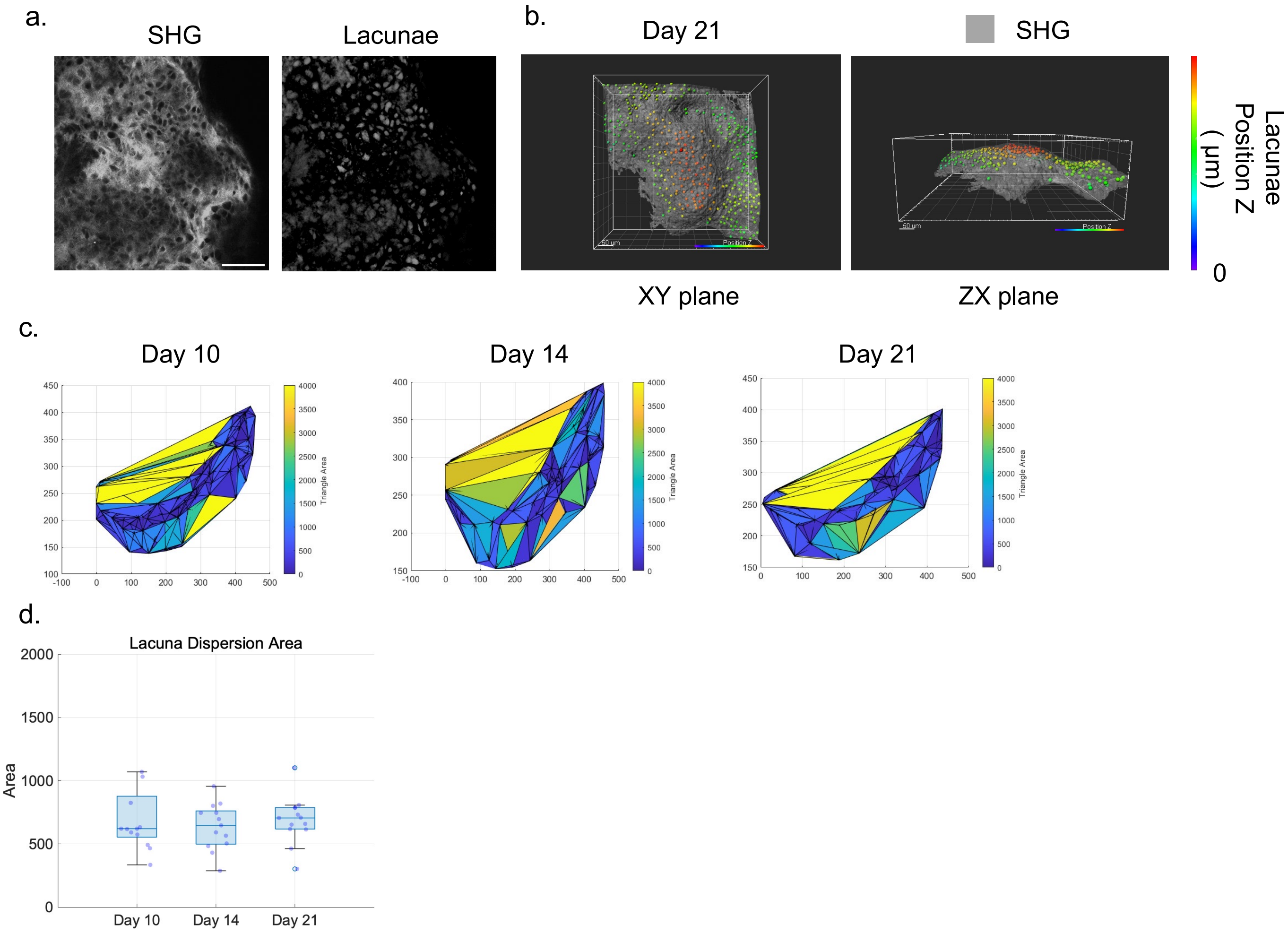

Extraction of Lacunae Structures from SHG images and Quantification of Density
a.Representative images of the extraction of lacunae structures from SHG images.
b. Spot detection of lacunae structure by IMARIS.
c.Representative images of density quantification by delaunay triangulation for lacunae structures.
d. A boxplot of the mean areas of the triangles. N = 13 from three independent experiments.,
One-way ANOVA with Tukey's post-hoc multiple comparison test.

Supplementary Figure. 7.

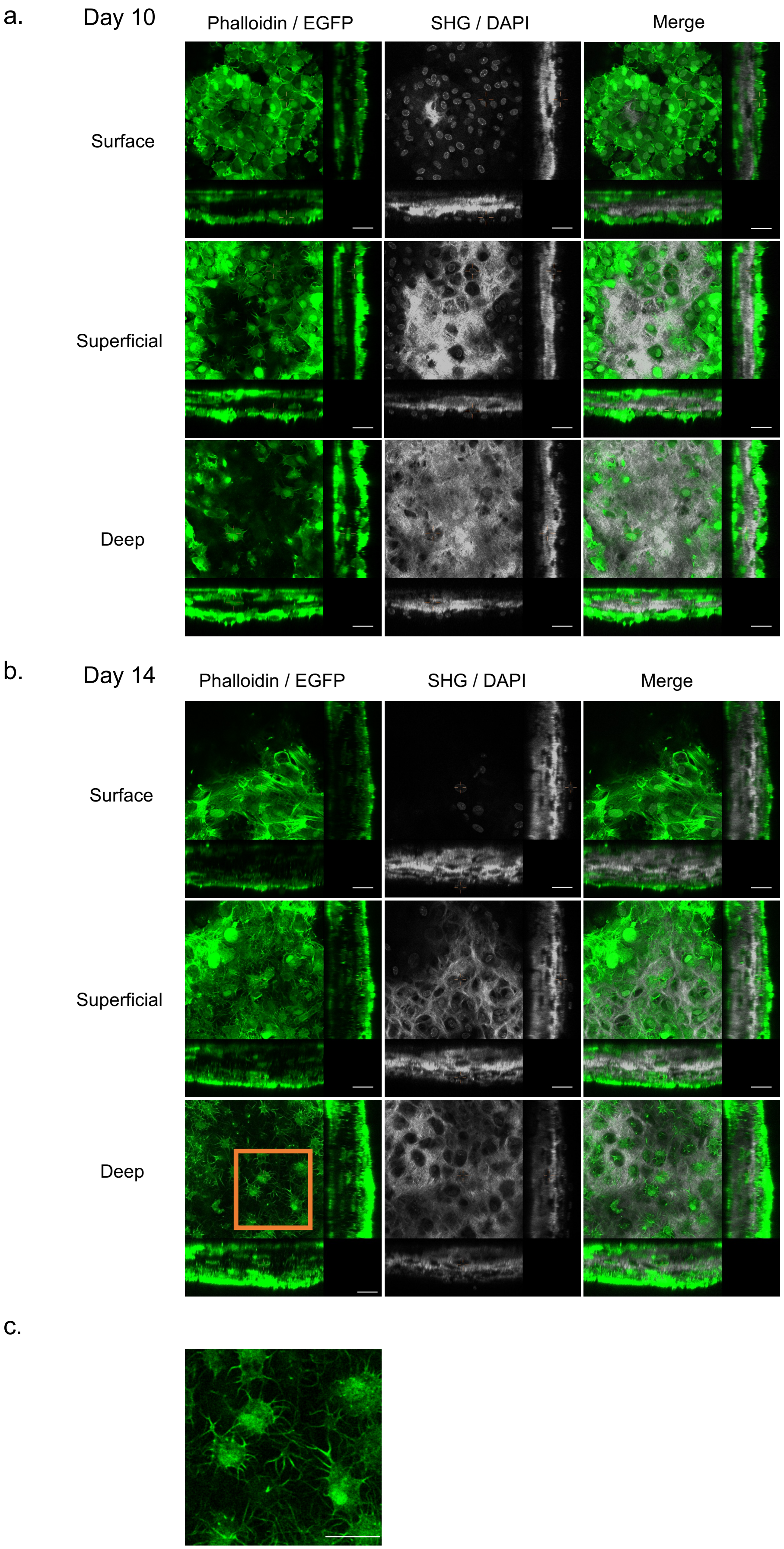

Phallodin and DAPI staining of bone nodules
a.Representative images of nodules on day 10. b.Representative images of nodules on day 14.
c. A cropped image from b.

Supplementary Figure. 7.

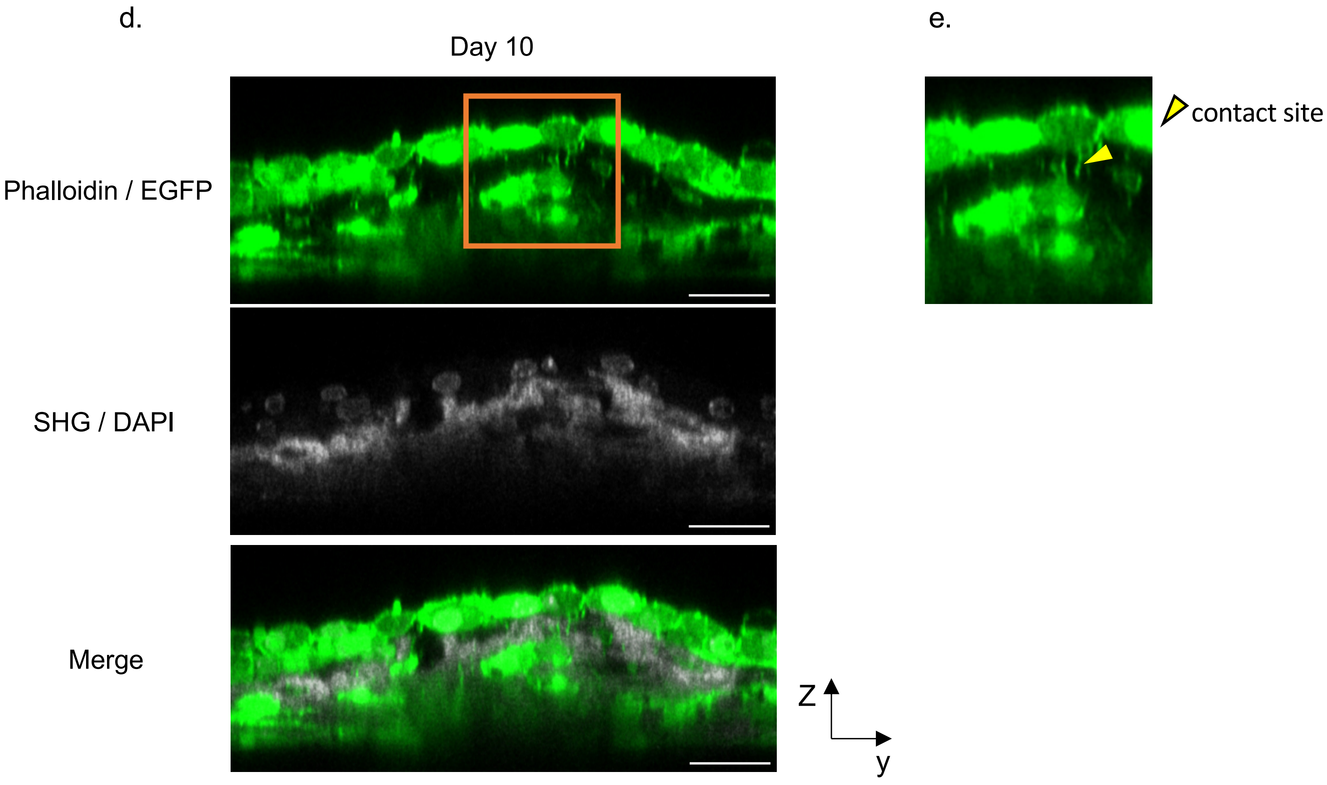

Phallodin and DAPI staining of bone nodules
d. Representative images of a bone-nodule in the YZ plane.
e. A croped image from c. (the yellow arrowhead indicated contact sites of osteocytes and osteoblasts.) ,
Scale bar : 25μm.

Supplementary Figure. 8.

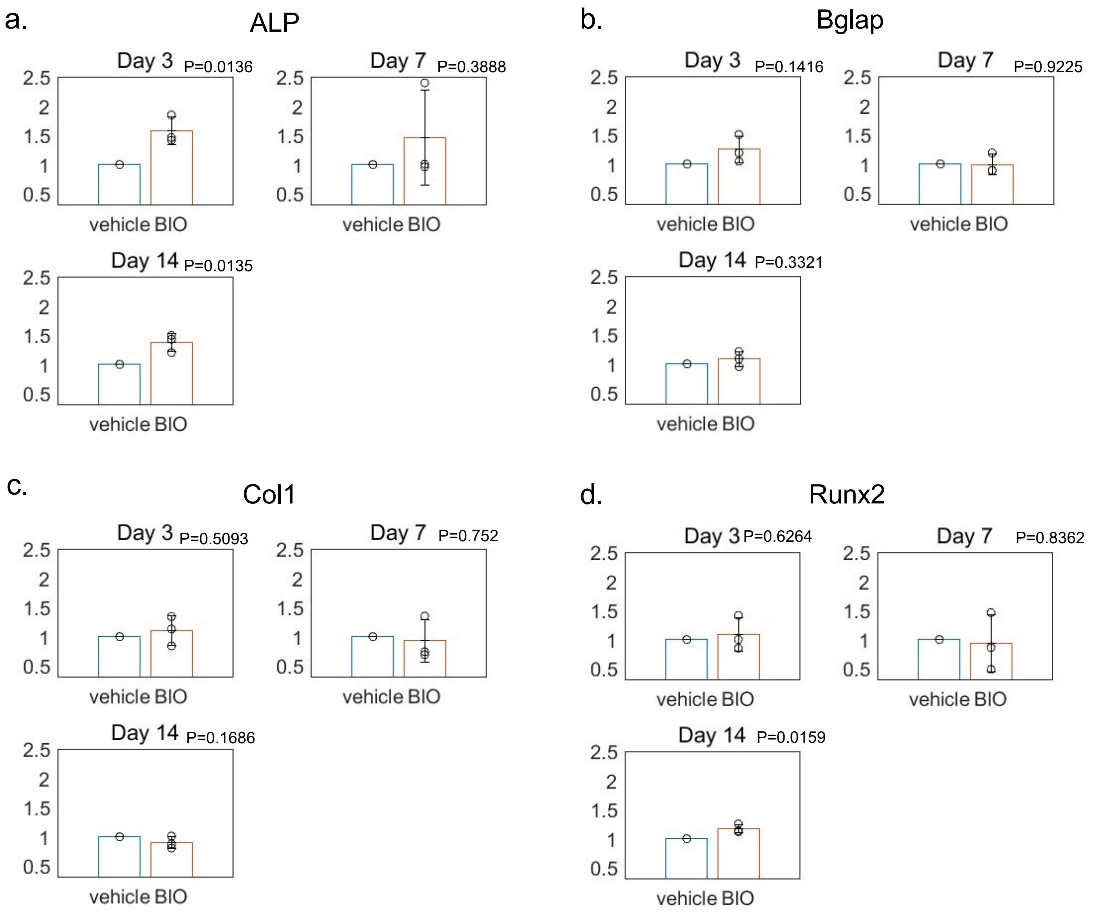

RT-qPCR of osteogenic markers
a.ALP b.Bglap c.Col1 d. Runx2.
N = 3, mean± SD.Statistical significance was determined by one-way ANOVA.

Supplementary Table. 1.

primers for qPCR

	Forwardw (5' → 3')	Revers (3' → 5')
ALP	GGGCGTCTCCACAGTAACCG	ACTCCCACTGTGCCCTCGTT
Bglap	CCAAGCAGGAGGGCAATA	AGGGCAGCACAGGTCCTAA
Col1	CCCCAACCCTGGAAACAGAC	GGTCACGTTCAGTTGGTCAAAGG
Runx2	GAGTCAGATTACAGATCCCA	TGGCTCTTCTTACTGAGAGA
GAPDH	ATGTGTCCGTCGTGGATCTG	TGAAGTCGCAGGAGACAACC
